# Göttingen Minipigs as a Model to Evaluate Longevity, Functionality, and Memory of Immune Response Induced by Pertussis Vaccines

**DOI:** 10.3389/fimmu.2021.613810

**Published:** 2021-03-17

**Authors:** Céline Vaure, Véronique Grégoire-Barou, Virginie Courtois, Emilie Chautard, Cyril Dégletagne, Yuanqing Liu

**Affiliations:** Research and External Innovation, Sanofi Pasteur, Marcy l'Etoile, France

**Keywords:** Göttingen minipigs, longitudinal study, pertussis vaccines, antibody response, T helper polarization, memory cells, single cell RNA sequencing

## Abstract

Evaluation of the short-term and long-term immunological responses in a preclinical model that simulates the targeted age population with a relevant vaccination schedule is essential for human vaccine development. A Göttingen minipig model was assessed, using pertussis vaccines, to demonstrate that vaccine antigen-specific humoral and cellular responses, including IgG titers, functional antibodies, Th polarization and memory B cells can be assessed in a longitudinal study. A vaccination schedule of priming with a whole cell (DTwP) or an acellular (DTaP) pertussis vaccine was applied in neonatal and infant minipigs followed by boosting with a Tdap acellular vaccine. Single cell RNAsequencing was used to explore the long-term maintenance of immune memory cells and their functionality for the first time in this animal model. DTaP but not DTwP vaccination induced pertussis toxin (PT) neutralizing antibodies. The cellular immune response was also characterized by a distinct Th polarization, with a Th-2-biased response for DTaP and a Th-1/Th-17-biased response for DTwP. No difference in the maintenance of pertussis-specific memory B cells was observed in DTaP- or DTwP-primed animals 6 months post Tdap boost. However, an increase in pertussis-specific T cells was still observed in DTaP primed minipigs, together with up-regulation of genes involved in antigen presentation and interferon pathways. Overall, the minipig model reproduced the humoral and cellular immune responses induced in humans by DTwP vs. DTaP priming, followed by Tdap boosting. Our data suggest that the Göttingen minipig is an attractive preclinical model to predict the long-term immunogenicity of human vaccines against *Bordetella pertussis* and potentially also vaccines against other pathogens.

## Introduction

Although the mouse is traditionally the species of choice for preclinical evaluation of vaccines, the results have limited translatability to humans since they do not always predict results in humans (1, 2). To overcome the limitation of using only mice, non-human primates (NHPs) can be used. But ethical and financial concerns, and the availability of suitable animal facilities for NHPs, limit their use in preclinical studies. Also it is difficult to perform suitable-sized NHP studies to evaluate pediatric vaccines in neonatal and infant animals. There are as well differences in vaccine responses between humans and NHPs, notably in newborns (3).

Pigs, and more particularly minipigs, offer many advantages as an alternative model for preclinical vaccine evaluation as they can be housed in large groups in standard animal facilities, despite being large animals. Delivery routes commonly used in humans can be used; and large volumes of blood can be drawn, unlike in mice (4, 5). Pigs are a large animal model that can be used to evaluate vaccines in special populations such as in neonates (5). Mimicking pediatric primary series followed by adolescent booster vaccination is more practical in minipigs than NHPs because pigs have relatively large litters; and are considered to be neonates to infant before 1 month of age and adolescent between 3 and 5 months of age, whereas NHPs are considered to be adolescent between 3 and 4 years of age (6). Longitudinal follow up of the immune response can be performed in the same individual animals, unlike in mice. The pig immune system is probably the best characterized system, after murine and primate immune systems and its development is very similar to that of humans (4, 7–9). The porcine immune system has been reported to be closer to humans than the mouse system. For example, the conservation of many immune-related gene families has been reported to be three-times greater, in terms of synteny, between humans and pigs than between humans and mice. The overall identity with human immune proteins is significantly higher in pigs than in mice and there is an increasing number of tools available to conduct immunological studies in pigs (10, 11). Minipigs have been used as an immunosafety model, showing good similarity with effects seen in humans, suggesting a good translatability to humans (7).

The objective of this study was to evaluate minipigs as a preclinical model for assessing short-term and long-term vaccine immunogenicity, using pertussis vaccines as an example.

Introduction of whole cell pertussis vaccine (wP) combined with diphtheria and tetanus antigens (DTwP) in the 1940's, followed by a less reactogenic acellular pertussis vaccine (aP) also combined with diphtheria and tetanus antigens (DTaP) in the 1990's led to a drastic decline in the incidence of reported cases of whooping cough. Despite of the fact that both wP and aP vaccines elicit an antibody response in vaccinees, DTwP or DTaP vaccines do not elicit the same profile of immune responses. For instance, DTwP induces T helper (Th)-1/Th-17 effector cells whereas DTaP induces Th-2 cells (12). Moreover, some reports suggest a difference in the longevity and memory of responses elicited by DTwP and DTaP vaccines (13).

We compared the humoral and cellular immune responses in Göttingen minipigs after priming with DTwP or DTaP, followed by a boost with modified acellular Tdap vaccine, 3 months later. The antibody response was monitored by Meso Scale Discovery (MSD) technology, functional antibodies by a pertussis toxin (PT) neutralization assay, and the Th profile and memory B cells by FLUOROSPOT. Finally, we, for the first time, used single cell RNA sequencing (scRNAseq) of minipig peripheral blood mononuclear cells (PBMCs) to phenotype the immune cells and explore their long term functionality after immunization. The results from these various evaluations provided a comprehensive, long-term study of the immune response elicited by the pertussis vaccines in the Göttingen minipig.

## Materials and Methods

### Vaccines

The whole cell pertussis vaccine (DTwP; D.T.COQ/D.T.P®, Sanofi-Pasteur Ltd.) contained ≥4 I.U. of heat-inactivated *Bordetella pertussis* (Bp), ≥60 I.U. of tetanus toxoid (TT) and ≥30 I.U of diphtheria toxoid (DT). The five-component acellular pertussis vaccine (DTaP; DTaP5cp-IPV-Hib, PEDIACEL®, Sanofi-Pasteur Ltd.), contained 20 μg chemically-detoxified pertussis toxin (PTxd), 20 μg filamentous hemagglutinin (FHA), 3 μg pertactin (PRN), 5 μg fimbriae 2/3 (FIM), 5 Lf TT, 15 Lf DT and 1.5 mg aluminum phosphate per dose. The modified acellular pertussis booster vaccine (Tdap) contained 10 μg PTxd, 5 μg FHA, 5 μg PRN, 7.5 μg FIM, 5 Lf TT, 2 Lf DT and 1.5 mg aluminum phosphate per dose.

### Animals

Pregnant Göttingen minipig sows were purchased from Ellegaard Göttingen Minipigs A/S and their piglets were born in Sanofi Pasteur's animal facilities. Piglets were labeled individually using transponders and visual identification. They were housed with their mother until weaning on D36. Males and females were then housed separately. This project was approved by Sanofi Pasteur's animal ethics committee and complied with European guidelines for animal care.

### Study Schedules

**Figure 1:**
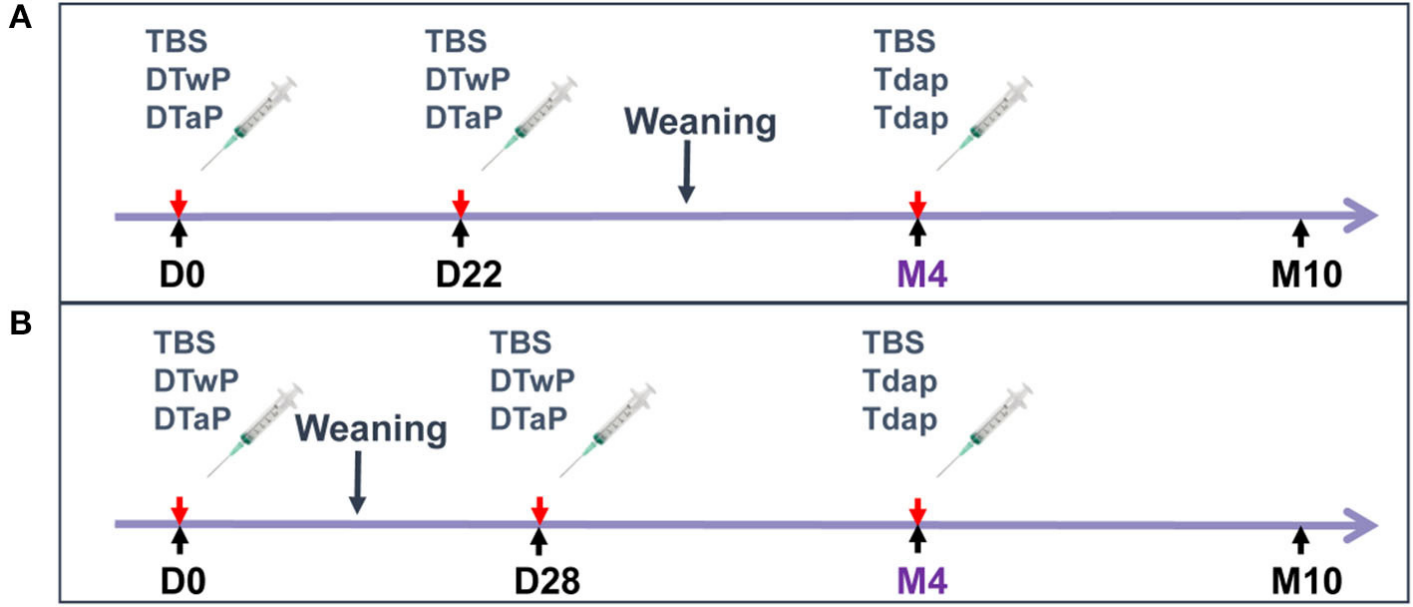
Study design. **(A)** First study: 4–6-day-old neonate minipigs from three different litters received intramuscular (IM) injections of DTaP or DTwP at ½ human dose (HD) or TBS (seven animals per group), followed by a second IM injection of 1HD of DTaP, DTwP or TBS on D22 and a booster injection with 1 HD of Tdap or TBS 3 months later (M4). **(B)** Second study: 3-week old infant minipigs from four different litters received an IM injection with 1 HD of DTaP or DTwP or TBS (five animals per group), followed by a second IM injection of 1 HD of DTaP or DTwP or TBS 4 weeks later (D28) and a booster injection with 1 HD of Tdap or TBS at month 4 (M4).

In the first study, 4–6-day old neonate minipigs from three different litters received an intramuscular (IM) injection of either DTaP or DTwP at ½ human dose (HD) (seven animals per group) (Figure 1A). A second injection of 1HD of DTaP or DTwP was administered on D22. Both groups were boosted with 1 HD of Tdap 3 months later (M4). A negative control group of three minipigs received three injections of tris buffer saline (TBS). Minipigs of different sexes and litters were allocated to the groups to minimize any genetic or gender effect on immune response to vaccination (14, 15).

In the second study, in which memory B cell assessments and scRNAseq analyses were performed, 3-week old infant minipigs from four different litters received an IM injection with 1 HD of either DTaP or DTwP (five animals per group) (Figure 1B). A second injection of 1 HD of DTaP or DTwP was administered 4 weeks later (D28). Both groups were boosted with 1 HD of Tdap at month 4 (M4). A negative control group of three minipigs received TBS on D0, D28 and M4.

### Blood Samples

Blood samples were collected from the cranial vena cava under anesthesia with IM Zoletil 50® (0.2 mL/kg) in serum-separation tubes (BD Vacutainer # BD366882) for antibody assessment or in sodium heparin plasma tubes (BD Vacutainer # BD368480) for PBMC isolation for the analysis of T cell and memory B cell FLUOROSPOT and scRNAseq assays.

### Analytical Assays

#### IgG Assay

Antigen-specific Immunoglobulin G (IgG) titers were determined using MSD electrochemical luminescence (MSD-ECL) assays according to the manufacturer's instructions. The MSD-ECL plates were printed with FHA, FIM2/3, PRN, PTxd, TT, DT antigens (multi-spot® 96-well-7 spot plates) by the manufacturer (Meso Scale Discovery). Serum IgG was detected using goat anti-pig IgG (Fc) biotin conjugate (Biorad #AAI41B) followed by Streptavidin-Sulfo-Tag® (MSD #R32AD-1).

#### Pertussis Toxin Neutralization Antibody Titers

PT neutralizing antibody titers were assessed in a Chinese hamster ovary (CHO) cell assay, as previously described (16). Briefly, CHO cells were incubated for 48 h with a mixture of sera and PT (pre-incubated for 2–3 h). CHO cells were then fixed with methanol and stained with Giemsa before reading the cell clusters. Samples with titers below the assay cut-off of 20 were arbitrarily attributed a titer of 10 (50% of the cut-off).

#### Memory B Cell FLUOROSPOT

PBMCs were stimulated for 4 days with R848 (InvivoGen #tlrl-r848-5) and recombinant porcine IL-2 (R&Dsystems #652-P2) and then incubated for 5 h on ELISPOT plates (Millipore #S5EJ104I07) previously coated with a pool of pertussis antigens (PTxd, PRN, FIM2/3, FHA), purified anti-porcine IgG mAb MT421 (Mabtech #3151-3-250) as positive control or medium alone as negative control. Memory B cells were detected using biotinylated anti-porcine IgG mAb MT424 (Mabtech # 3151-6-250) followed by streptavidin-PE (Southern Biotech #7100-09L). Positive spots were counted with an automatic ELISPOT fluorescent plate reader (Microvision). Data shown after correction for background by subtraction of the counts from negative control wells.

#### T Cell FLUOROSPOT

ELISPOT plates (Millipore #S5EJ104I07) were coated with mouse anti-pig IFN-γ (ThermoFisher #MP700), goat anti-porcine IL-4 (R&Dsystems #AF654), or rabbit anti-swine IL-17A (KingFisher Biotech #PB0158S-100). After blocking with RPMI medium supplemented with glutamine, streptomycin, penicillin, β mercaptoethanol and 10% fetal bovine serum, PBMCs were added and stimulated for 48 h with a pool of pertussis antigens (PTxd, PRN, FIM2/3) or heat inactivated whole cell *B. pertussis* (hk*Bp*). IFN-γ, IL-4 and IL-17 secreting cells were detected using biotin mouse anti-pig IFN-γ (BD Biosciences #559958), goat anti-porcine IL-4 (R&Dsystems #BAF654) and rabbit anti-swine IL-17A (KingFisher Biotech #PBB0270S-050), respectively, followed by streptavidin-PE (Southern Biotech #7100-09L). Data shown after correction for background by subtracting count from negative control wells.

#### Single Cell RNA Sequencing (scRNAseq)

PBMCs from control minipigs and DTaP-primed minipigs were incubated at +37°C for 16 h in a 5% CO_2_ atmosphere with PBS (*n* = 2 per group) and PBMCs from DTaP-primed minipigs were incubated with a pool of pertussis antigens (*n* = 2). Cells were washed with PBS and the number of live cells was determined with a NucleoCounter NC 200 (Chemometec). The cells were resuspended in PBS with 0.04% BSA to obtain an expected cell recovery population of 10,000 cells per channel, loaded on 10X chip and run on the Chromium Controller system (10XGenomics) to be used directly for single-cell sequencing. scRNAseq libraries were generated with the Chromium Single Cell 3′ v3 assay (10X Genomics, PN-1000075) and sequenced on the NovaSeq 6000 platform (Illumina, S2 flowcell, # 20012862) to obtain around 30,000 reads per cell.

##### Sample Preprocessing

The Cellranger Single-Cell Software Suite (v3.0.2) was used to perform sample demultiplexing, alignment to the closest available genome, the pig genome (Sscrofa11.1, Ensembl release 98), barcode assignment for each cell, and gene counting by unique molecular identifier (UMI) counts. Data were analyzed in RStudio (v1.1.383, R v3.6.1) using the Seurat (v3.1.0) package (17). Only cells with at least 500 molecules, 200 unique genes detected, and <20% of reads that mapped to the mitochondrial genome were used. Data were normalized using the scran package (v1.12.1) (18). Variable genes were determined for each sample using Seurat's FindVariableGenes function with default parameters and merged using Seurat integration strategy based on “anchors.” Scaled values were calculated using ScaleData function with default settings.

##### Clustering

Principal component analysis (PCA) was performed on the first 60 principal components (PCs). Clusters in the resulting subspace were then identified using the FindClusters function with the resolution set to 0.6 and mapped using the RunUMAP function. Specific marker genes for clusters were identified using the FindAllMarkers function to identify cell phenotypes. Clusters containing all activated T cells were split according to CD8A, CD8B and CD4 expression to enable CD4 and CD8 activated T cells to be distinguished. A similar strategy based on CD2 expression was used to isolate γδ non-terminally differentiated T cells from other γδ T naive cells. Cell phenotypes were checked by comparing the mean expression of key marker genes in each cell type with the overall mean expression and results were visualized using the corrplot R package (v0.84).

##### Differential Gene Expression and Functional Enrichment Analysis

Gene expression under different experimental conditions for each cell type was assessed using Seurat FindMarker function, and analyzed using Wilcoxon rank testing. Only genes with absolute log-fold changes higher than 0.2 and adjusted *P*-value <0.05 were retained. Ortholog pig and human genes were determined using the BiomaRt R package (v2.40.3), keeping only 1-to-1 orthologs for which only one copy was found in each species, and *Sus scrofa* gene names were replaced by their corresponding human Ensembl ortholog identifiers before being processed in Ingenuity Pathway Analysis (IPA) software using default parameters (19, 20).

### Statistical Analysis

The normal distribution of log-transformed data was confirmed before analysis. Differences between TBS- and DTaP- or DTwP-primed groups, as well as differences between DTaP- and DTwP-primed groups were assessed with one-way analysis of variance (ANOVA). Analyses were not performed when there were more than 50% of non-responders. The residuals were assessed to verify the model's validity. All analyses were done on SAS v9.4®. Differences were considered significant at a *p*-value < 0.05.

## Results

### Antigen Specific IgG Responses Can Be Monitored in Minipigs Immunized With DTaP, DTwP and Tdap Vaccines

**Figure 2:**
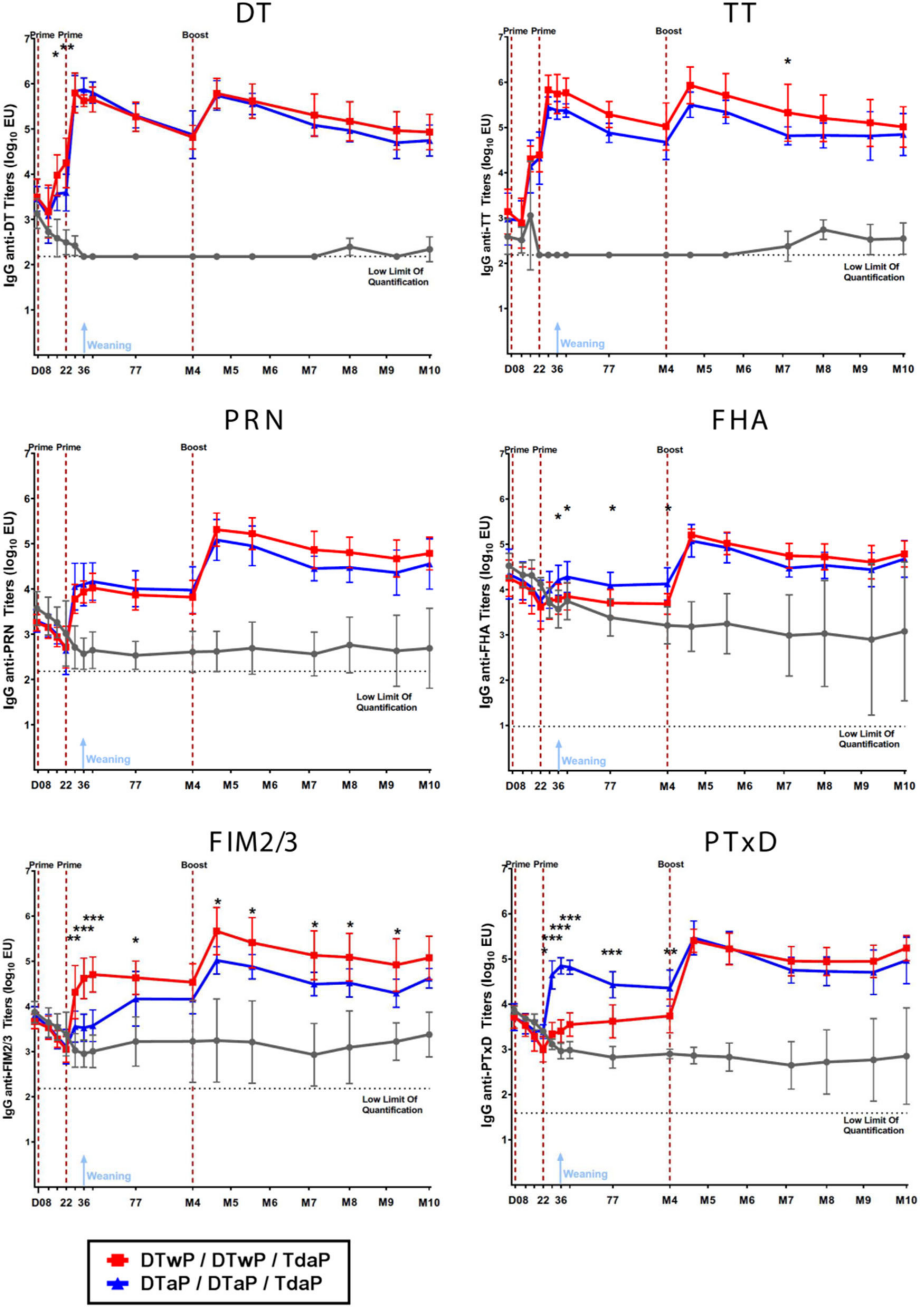
IgG titers against DT, TT, PRN, FHA, FIM2/3 and PT measured by the MSD-ECL assay in neonatal minipigs primed with either DTwP (*n* = 7) or DTaP (*n* = 7) and boosted with Tdap vaccine and in control (TBS-injected) minipigs (*n* = 3). Data are shown as log_10_ geometric mean titers with standard deviations. *p-value < 0.05, **p-value < 0.01, and ***p-value < 0.001 for comparison between the DTaP- and DTwP primed groups.

Both DTaP- and DTwP-priming induced statistically-significant IgG responses against non-pertussis antigens (TT and DT) after the first injection and against the pertussis antigens (PT, PRN, FIM2/3, FHA) after the second injection, as compared to the TBS control group (Figure 2). DTaP priming induced a significantly more rapid and stronger anti-PT IgG response (*p* < 0.001), and to a lesser extent, the anti-FHA IgG response (*p* < 0.05), whereas DTwP priming induced a more rapid and stronger anti-FIM IgG response (*p* < 0.05 or < 0.001, depending on the time-point). The anti-PRN IgG responses were similar in the two groups. A boost by Tdap increased the IgG titers against all antigens in both DTaP and DTwP primed groups.

### PT Neutralizing Antibodies Were Measured Post-boost in DTaP- but Not DTwP-Primed Minipigs

**Figure 3:**
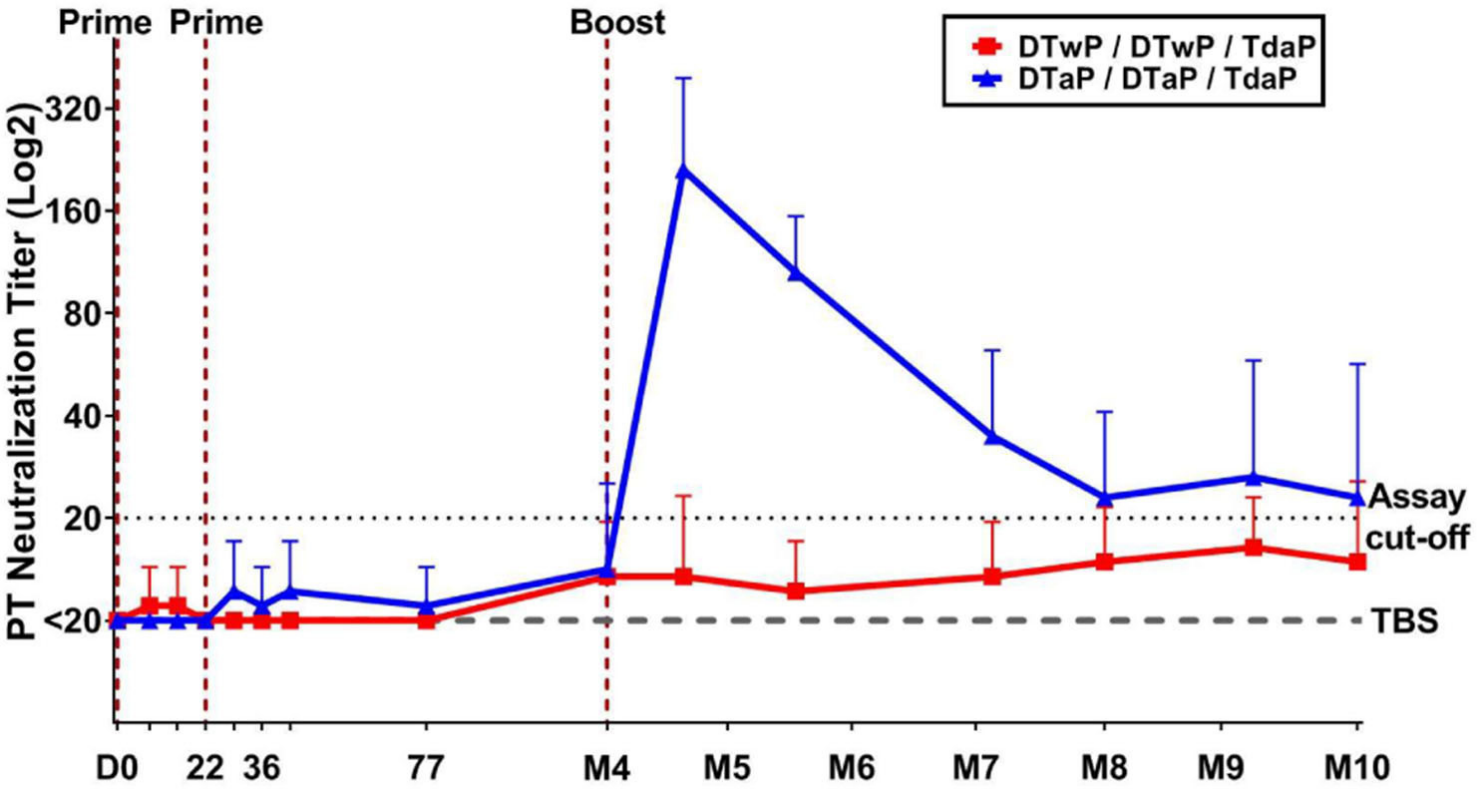
Functional antibodies measured by PT neutralization assay in neonatal minipigs primed with either DTwP (*n* = 7) or DTaP (*n* = 7) and boosted with Tdap vaccine. Data are shown as geometric mean titers with standard deviations for the DTwP and DTaP groups. The geometric mean titer for the control group (*n* = 3) that received TBS injections is shown (dashed line). All values below the assay cut-off were arbitrarily attributed a titer of 50% of the cut-off value.

To evaluate the functionality of pertussis-specific antibodies, PT neutralizing antibodies were assessed. Two vaccinations in neonatal minipigs (before weaning) induced no detectable PT neutralizing antibody in either group (Figure 3). After boosting minipigs with Tdap, PT neutralizing antibodies were detected in those primed with DTaP, but not in those primed with DTwP. The PT neutralizing antibodies peaked 1 month after the Tdap boost and decreased to a low level at M8 and then stabilized for the following 2 months. PT neutralization titers were below the limit of detection in the control (TBS) group. No statistical analyses were performed between groups because of the high number of non responders in the TBS and DTwP groups.

When 3-week old infant minipigs were primed, the induction of the IgG and PT neutralization responses was more rapid than in neonatal minipigs (data not shown). Particularly, the first prime injection induced IgG responses against all antigens. PT neutralizing antibodies were induced after the 2nd prime in DTaP-primed minipigs, reaching a geometric mean titer (GMT) of 90 on day 42 and 640 at 3-weeks post-Tdap boost, compared with GMTs of <20 and 210, respectively, in neonatal DTaP-primed minipigs. The induced PT neutralization response reached a titer of 120 from M5 to M7 after the Tdap boost in DTwP-primed minipigs.

### Pertussis-Specific Memory B Cells Were Detected Up to at Least 6 Months Post-Tdap Boost

**Figure 4:**
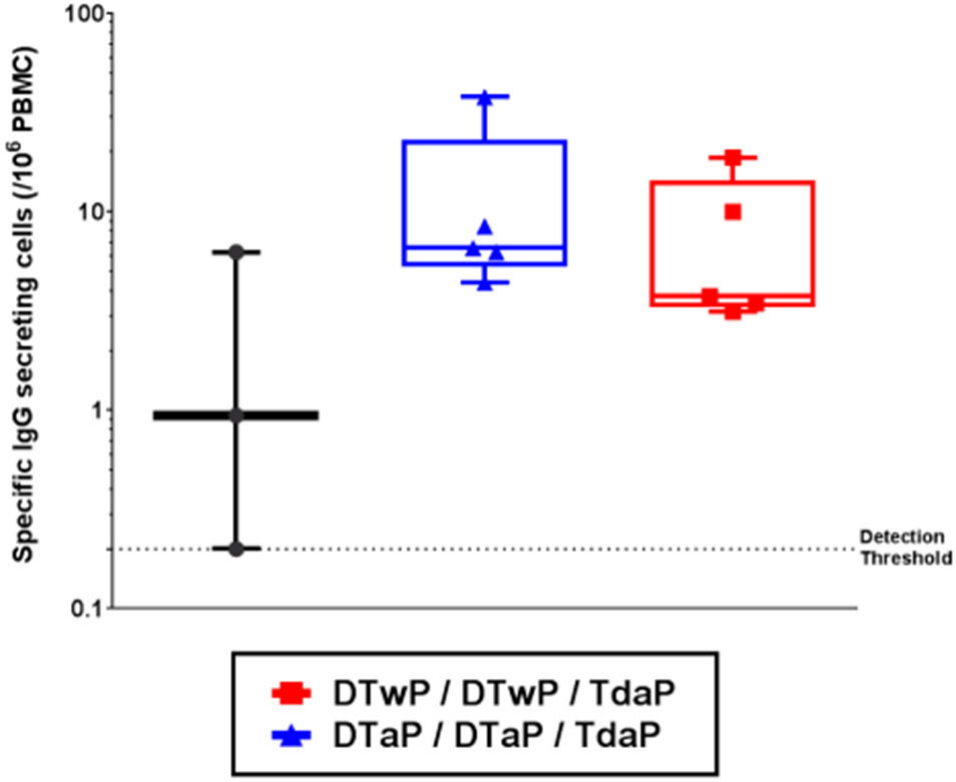
Memory B cells specific for a pool of pertussis antigens (PTxd, PRN, FIM2/3, FHA) measured by FLUOROSPOT 6 months post-Tdap boost in infant minipigs primed with either DTwP or DTaP vaccine (first dose at 3-weeks old) and boosted with Tdap vaccine. Data shown as box and whisker plots with median and min/max (*n* = 5 for DTwP and DTaP groups and *n* = 3 for TBS group).

The pertussis-specific circulating memory B cells were assessed in the minipigs that received their first dose of vaccine at 3-weeks old. Memory B cells, specific for a pool of pertussis antigens (PTxd, PRN, FIM2/3, FHA) were found to persist at a low but detectable level 6 months after the Tdap boost (Figure 4). No statistical difference was found between DTwP- and DTaP-primed groups.

### DTaP and DTwP Priming Induced Th-2 and Th-1/Th-17 Biased Profiles, Respectively, That Were Sustained After a Tdap Boost

**Figure 5:**
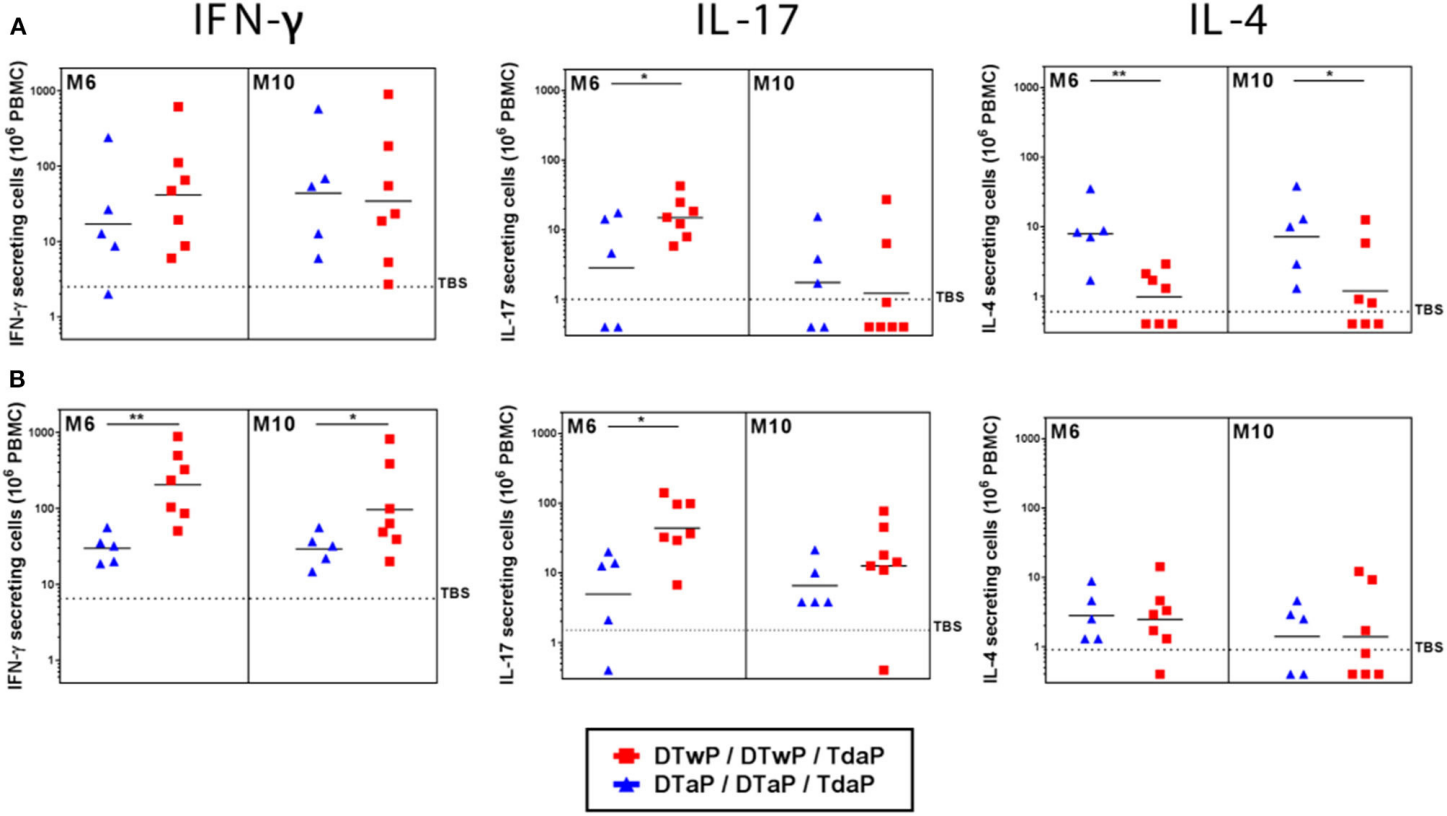
IFN-γ (Th-1), IL-17 (Th-17) and IL-4 (Th-2) secreting cells measured by FluoroSpot assay at M6 and M10 on PBMCs from neonatal minipigs primed with either DTwP or DTaP and boosted with Tdap vaccine, stimulated for 48 h with **(A)** a pool of pertussis antigens (PTxd, PRN, FIM2/3) and **(B)** heat-killed B. pertussis (hk*Bp*). Data are shown for DTwP (*n* = 7) and DTaP (*n* = 7) groups as individual and geometric mean titers, after correction for background levels of cytokine secreting cells in the negative control wells (media alone). Data for the control group (TBS, *n* = 3) are shown as the geometric mean titer for all minipigs at all time-points. *p-value < 0.05 and **p-value < 0.01 for statistical comparison of DTaP- and DTwP primed groups.

To characterize the Th profile induced by DTaP- and DTwP-priming in the minipigs, the number of IFN-γ, IL-4, and IL-17 secreting cells were counted in a FluoroSpot assay to determine Th-1, Th-2, and Th-17 responses, respectively. DTaP-priming induced mainly a Th-2 biased response, whereas DTwP-priming induced a Th-1/Th-17 biased response to the pool of pertussis antigens (Supplementary Figure 1). This Th-2 vs. Th-1/Th-17 bias persisted after a Tdap boost (Figure 5A). While the IL-4 and IFN-γ responses to the pool of pertussis antigens were stable, the IL-17 response in the DTwP primed group appeared to be transient after Tdap boost, and decreased to baseline level at M10, that is, 6 months post boost.

Following stimulation with hk*Bp*, both the IFN-γ and IL-17 responses were significantly stronger in DTwP-primed minipigs than in DTaP-primed minipigs (*p* < 0.05), with a peak at M6 to M7, and little or no decrease up to M10 (Figure 5B). The frequency of IL-4 secreting cells was at a similarly low level in both the DTwP- and DTaP-primed groups.

In addition, we observed a consistent Th-2 profile in the DTaP-primed infant minipigs, with a higher level of response (geometric mean titers between 30 and 70 IL-4 secreting cells/10^6^ PBMCs) than in the DTaP-primed neonatal minipigs. However, we observed a low IFN-γ response in the DTwP-primed group and a few to undetectable number of IL-17-secreting cells in infant minipigs in both the DTwP- and DTaP-primed groups.

### Pertussis-Specific T Cells Were Detected 6 Months Post-Tdap Boost in DTaP-Primed Minipigs by scRNAseq

We used single cell RNA sequencing (scRNAseq) on a 10X Genomics platform to phenotype minipig PBMCs 6 months post-Tdap boost (M10), when T cell and memory B cell FluoroSpot assays were also performed. The final dataset that passed quality control contained 15,286 cells from the TBS control group incubated with medium, 15,189 cells from the DTaP-primed group incubated with medium and 18,421 cells from the DTaP-primed group incubated overnight with the pool of pertussis antigens.

**Figure 6:**
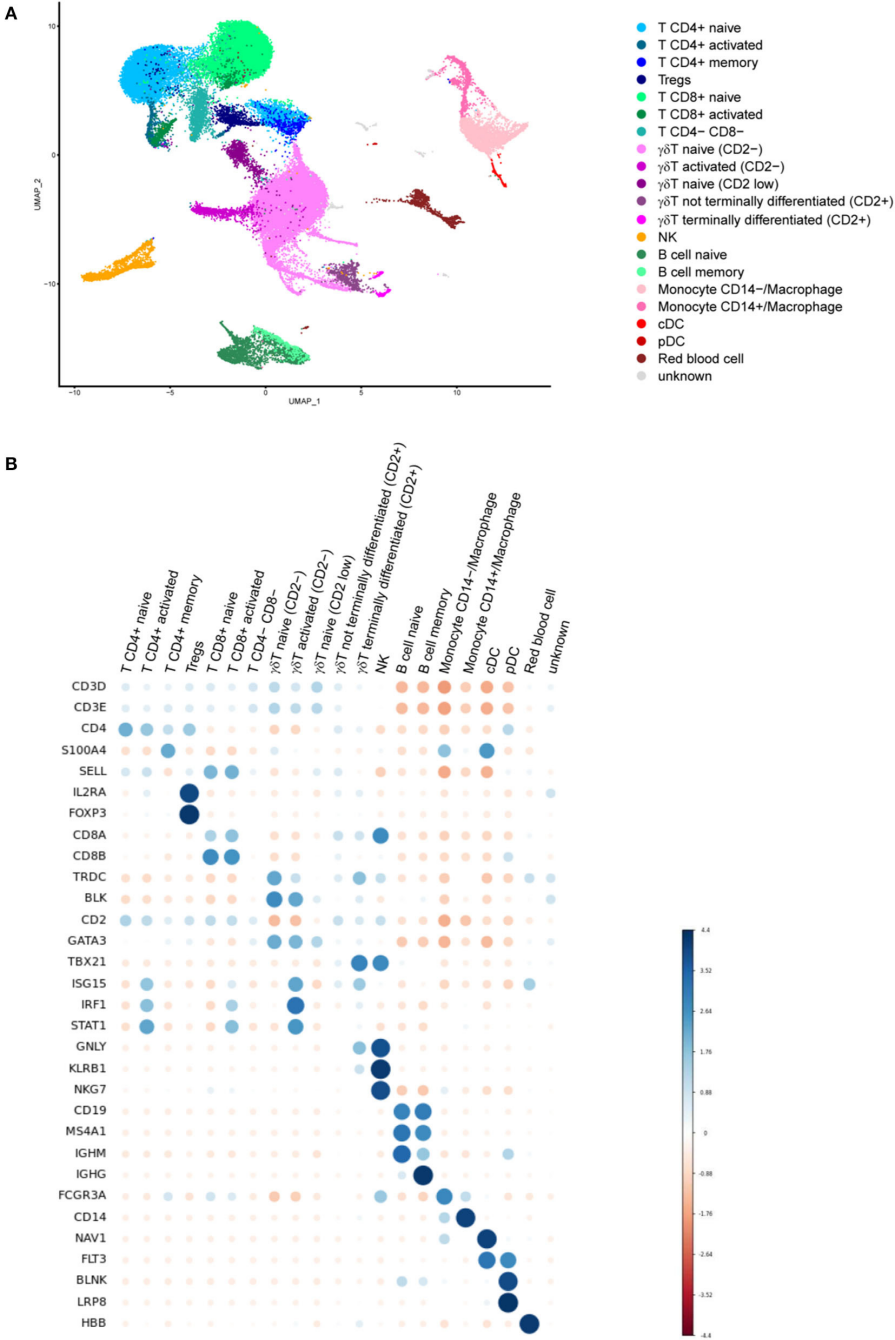
**(A)** Uniform manifold approximation and projection (UMAP) plot showing the results of single cell RNA sequencing (scRNAseq) of minipig PBMC populations. The plot shows scRNAseq results for PBMC immune cells from control minipigs incubated with medium (*n* = 2) and from DTaP-primed minipigs incubated with medium (*n* = 2) or a pool of pertussis antigens (PTxd, PRN, FIM2/3) (*n* = 2). **(B)** Dot plot showing the expression marker genes in each cell population. Blue dots indicate that the expression was higher than the average expression in the entire dataset in this population and red dots indicate that it was lower (MS4A1 = CD20 and FCGR3A = CD16A).

The analysis showed 30 different clusters composed of major immune cell types including CD4^+^, CD8^+^and γδ T cells (21, 19, and 34% of all isolated cells, respectively), monocytes (8%) and dendritic cells (DCs, 0.5%), as well as B cells (6%) and natural killer cells (NK, 3.5%) (Figure 6A). These populations were identified by their specific gene signature as CD3D and CD3E (T cells), NKG7 (NK cells), MS4A1 i.e., CD20 (B cells), CD14 and FCGR3A i.e., CD16A (monocytes) or FLT3 (DCs) (Figure 6B). T cell populations were split according to the expression of CD4 (T CD4^+^), CD8B and CD8A (T CD8^+^) as well as TRDC and BLK (γδ T cells). Combinations of specific markers were then used to distinguish subpopulations in CD4^+^ T cells (naïve, memory S100A4^+^/SELL, regulatory FOXP3^+^/IL2RA^+^), γδ T cells (naïve with no or low CD2 expression, non terminally differentiated GATA3^−^/CD2^+^, as well as terminally differentiated TBX21^+^), B cells (naïve IGHM^+^, memory IGHG^+^), monocytes (CD14^+^ or CD14^−^) and DCs (plasmacytoid DC BLNK^+^/LRP8^+^, and conventional DC NAV1^+^) (Figures 6A,B). Our analysis revealed a high expression of IRF1, ISG15 and STAT1 in some specific clusters of T cell subpopulations that were, therefore, categorized as “activated.”

**Figure 7:**
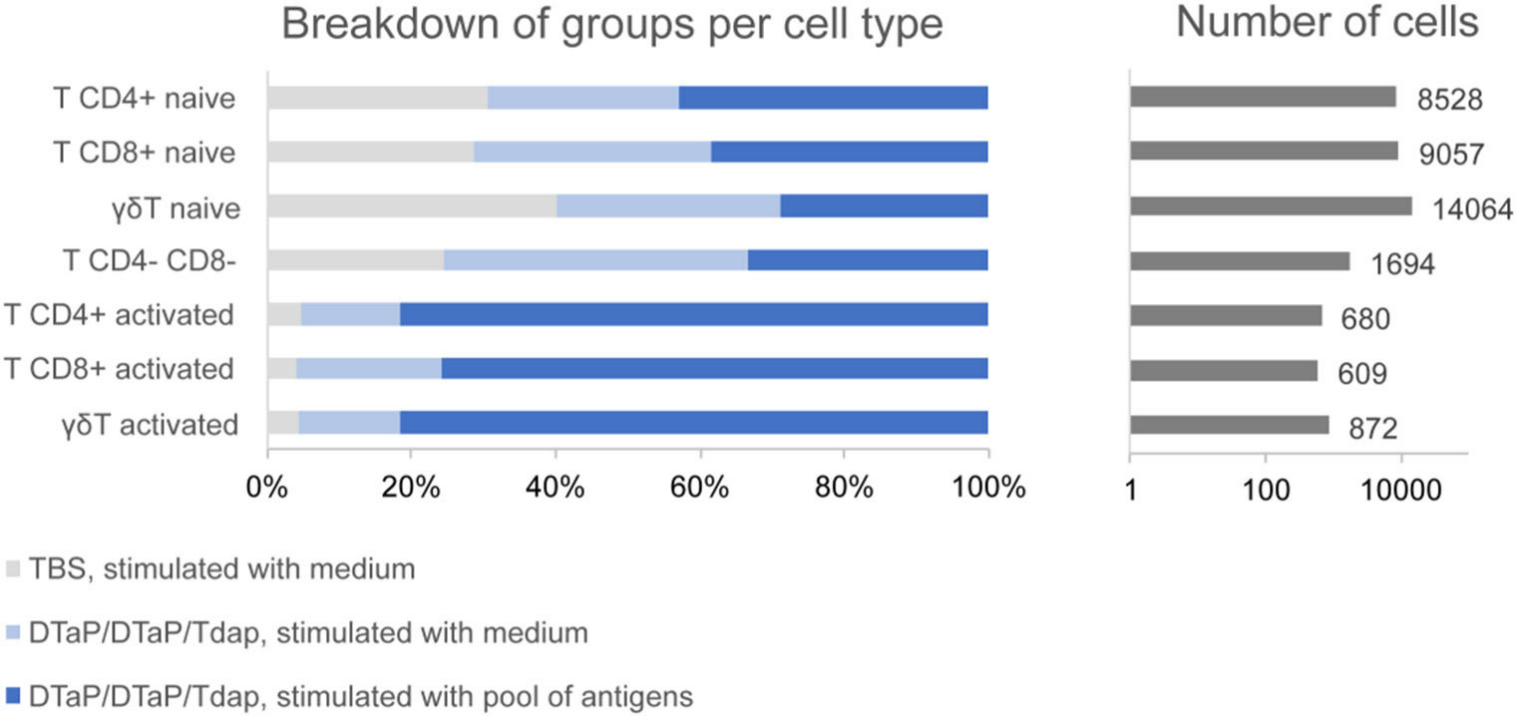
Group distribution of activated and non-activated T cell subpopulations within the entire set of T cells obtained by scRNAseq for the control TBS group incubated with medium (*n* = 2), DTaP-primed group stimulated with medium (*n* = 2) and DTaP-primed group stimulated with a pool of pertussis antigens (PTxd, PRN, FIM2/3) (*n* = 2).

Cell frequencies within a population did not appear to change drastically in the three groups analyzed, but no statistical test was performed to compare between the groups due to the low number of individuals in each group (*n* = 2 per group). However, the numbers of activated T cells (CD4^+^, CD8^+^ and γδ T cells) were much higher in the samples from the DTaP-primed group incubated with the pool of pertussis antigens than in those from the DTaP-primed or TBS groups incubated with medium (Figure 7).

### Genes Involved in Antigen Presentation and Interferon Pathways Were Up-Regulated in DTaP Primed/Tdap Boosted Minipigs After *in-vitro* Stimulation With Pertussis Antigens

Gene expression within each cell population showed limited quantitative differences between the DTaP-primed group and the TBS control, both incubated with medium, and between the DTaP-primed group incubated with the pool of pertussis antigens and the DTaP-primed group incubated with medium (Supplementary Figure 2). In the first comparison, DTaP-primed vs. TBS control, the majority of genes were up-regulated, mainly in the NK cells and monocytes (51 and 35 upregulated genes, respectively), and to a lesser extent in B cells (21 upregulated genes). The results from the IPA analysis showed, in NK cells, a coordinated overexpression of genes involved in NK cell signaling (*p*-value adjusted by BH test = 0.002), such as KLRB1, KLRK1, NCR1 (NKp46), PIK3R1 or TYROBP in the DTaP-primed group compared with the TBS control group, when both were incubated overnight with medium. For the second comparison, with DTaP-primed groups incubated with the pool of pertussis antigens vs. medium, there was a majority of down-regulated genes. Among the up-regulated genes, the IPA analysis showed an activation of the antigen presentation pathway (*p*-value adjusted by BH test = 8E-5) in monocytes (CALR, CIITA, HLA-DRA, LMP2, TAP1) and a stimulation of the interferon pathway supported by an increase of STAT1 or IRF1 expression, amongst others, in monocytes, T and γδ T cells (*p*-value adjusted by BH test < 0.01). Down-regulated genes for each comparison did not appear to be linked with particular intra cellular pathways.

## Discussion

In this study, we evaluated the Göttingen minipig as a preclinical model for vaccine immunogenicity. This evaluation was based on common readouts such as vaccine antigen-specific IgG and functional antibody titers, the T helper cytokine profile and memory B cell response, as well as the emergent scRNAseq approach to investigate further in depth the immune response. We showed that IgG titers against DT, TT, FHA, FIM, PRN and PT and pertussis toxin neutralizing antibodies, can be measured in minipigs. The responses were shown to be similar, but stronger when the first vaccination dose was administered in infant rather than in neonatal minipigs.

Measurement of total IgG level against vaccine antigens is the only immune assessment accepted by regulatory authorities for licensing acellular pertussis vaccines. We evalutated the IgG titers against the pertussis and non-pertussis antigens in the acellular vaccines in the sera from minipigs. Elevated initial IgG antibody titers against DT, TT, FHA, FIM, PRN and PT were observed on D0 in the control TBS group, which gradually decreased during the first 40 days after birth. These elevated IgG antibodies might be partially due to maternal antibodies acquired by the neonates from colostrum. Due to the epitheliochorial structure of the porcine placenta, piglets are born agammaglobulinemic and depend on receiving antibodies from colostrum which is richer in immunoglobulins, notably IgG, than human colostrum (21, 22). Pertussis-specific antibodies (FHA, FIM, PRN, PT) were detected in the sow sera (data not shown), probably due to the exposure to *B. bronchiseptica* that was present in Ellegaard barriers. Down-selection of the sows free of *B.bronchiseptica* could be a solution to avoid interference by *B.bronchiseptica* carriage to the immune response, but this was not done in our studies. The high anti-PT IgG in the sows was an unexpected finding because *B. pertussis* is thought by many to be the only bacterial species to produce the pertussis toxin. Some authors have reported that *B. bronchiseptica* does not have a PT gene (23), while others have indicated that *B. bronchiseptica* has a PT gene but that it was probably not expressed in their experimental conditions (24–26). Evidence suggests that certain genes from pathogenic organisms may be transcriptionally silent during growth on a rich laboratory medium, but can subsequently be induced during infection (27). If the PT expression in *B. bronchiseptica* were to be induced during infection, it could explain the observed anti-PT IgG titers in sera as a result of an anti-*B. bronchiseptica* immune response in the sows. Nevertheless, the elevated IgG titers against DT and TT up to D36 in the piglets suggest that *B. bronchiseptica* cross-reactive maternal antibodies are not the only explanation. Background IgG due to maternal antibodies and cross-reactive antibodies in piglets have been reported, and this could also explain the background levels observed in our study for specific pertussis and non-pertussis IgG titers (28).

For all antigens, the IgG responses peaked 1 month post-Tdap boost, i.e., M5, followed by a decline over the time. Noticeably, the IgG antibodies fell rapidly during the initial couple of months after M5, depending on the antigen, before reaching a stable level. The IgG titers to all antigens remained higher than those measured prior to vaccination, 6 months post-Tdap boost. This pattern of non-linear IgG decline after pertussis vaccination is in agreement with the observations in humans and baboons (29–31). We observed differences in the onset of IgG responses against the pertussis antigens (PT, FHA, FIM, PRN) and the non-pertussis antigens (DT and TT). These differences were consistent with the fact that TT is a strong immunogen whereas FHA, FIM, PRN and PT antigens are relatively weak, and that the immunogenicity of DT is intermediate (32). In addition, presence of maternal antibodies may impact the immune response to priming vaccination. We observed that the higher the antibody titer against a pertussis-specific antigen in a sow, the lower the neonatal antibody response to this antigen in the piglets born to the sow (data not shown). This interference from maternal antibodies present at time of immunization (known as blunting) has been observed in neonatal mice as well as in humans (33–35). The ratio of the maternal antibodies to vaccine antigens determines the extent of the inhibition, because there may be sufficient B cell epitopes free from maternal antibodies to be bound by the infants' B cells (36).

Since PT is a major *B.pertussis* virulence factor and antibody-mediated neutralization of PT was reported to be essential to control pertussis disease, it was important to monitor the total anti-PT IgG and functional PT neutralizing antibodies (37). There was a stronger and faster total IgG response to PT in the DTaP-primed group than in the DTwP-primed group; but only the DTaP-primed neonatal minipigs mounted a functional PT neutralizing antibody response. In agreement, the propensity of higher anti-PT responses induced by DTaP than DTwP was reported in humans (38). This may be attributed to a higher PT content in DTaP than in DTwP vaccines (39). The PT concentration in the DTaP vaccine is well defined (20 μg/HD), whereas the PT concentration in the DTwP vaccine is batch specific and not documented but is estimated to be lower. In our studies, priming 4–6-day old neonatal minipigs with DTwP induced anti-PT IgG but no anti-PT functional antibodies at any time. In contrast, 3-week-old infant minipigs primed with DTwP produced the functional anti-PT antibodies after a Tdap boost. Moreover, a PT neutralizing response was induced after the 2nd priming dose in DTaP-primed 3-week-old infant minipigs but not in 4–6-day old neonatal minipigs. These data altogether suggest a better antibody maturation when immunization is performed in older piglets, in agreement with the notion of immature neonatal immune system (40–43). The possibly longer time required for neonatal immune system to fully develop in pigs and humans may partially explain the disparity observed in baboons in terms of the anti-PT antibody responses. In pre-adult baboon, DTwP induced similar or higher anti-PT IgG and PT neutralizing antibodies than DTaP (31). The anti-PT IgG and PT neutralizing antibodies peaked 1 month post-Tdap boost in the minipigs, and then waned over time, as previously described in humans (37).

In addition to the humoral response, the cellular contributors to the immune response are crucial to establish an efficient immune response against *B. pertussis* (12, 44). Th-1, Th-2 and Th-17 FluoroSpot assays were developed to investigate cellular immune responses. IFN-γ secreting cell measurement has been previously described in minipigs (45). But to the best of our knowledge, it is the first time that Th-1, Th-2 and Th-17 profiles were monitored simultaneously in the minipig model using FluoroSpot assays. Our assessment of the T cell immune response focused on the post-Tdap boost period to mirror the same period that Th responses have been measured in many human studies (46, 47).

The importance of T helper response in the immunity against *B.pertussis* infection and colonization has recently been reviewed (12). In our minipig model we observed a Th-1/Th-17 biased profile in DTwP-primed minipigs and a Th-2 biased profile in DTaP-primed minipigs, as previously described in humans and baboons (31, 48, 49). These characteristic Th profiles were observed after priming and persisted during the post-Tdap boost period, highlighting a potential imprinting effect of DTaP vs. DTwP priming in the minipig model, with a Th-2 biased response for DTaP and a Th-1/Th-17 biased response for DTwP, similar to that reported in humans and baboons (31, 46, 48). The observed Th-2 profile was similar in the studies with neonatal and infant primed minipigs unlike the Th-1 and Th-17 profiles that showed some differences. This could be caused by variabilities in the batches of DTwP vaccine used in the studies, or heterogeneity between the minipigs. Alternatively, the T cell FluoroSpot assay may not be sufficiently robust in minipigs with batch variations of anti-cytokine antibody reagents. Although, human neonates have prefentially Th-2 biased responses, the Th response in neonatal and infant minipigs has not been fully characterized, to the best of our knowledge (42).

IFN-γ, IL-4 and IL-17 were still detectable 6 months post-Tdap boost. Moreover, the Th-2 biased profile associated with DTaP priming, and the Th-1/Th-17 biased profile associated with a DTwP priming were still observed several months post-Tdap boost, as reported in humans (46). Thus, the minipig model can be used to evaluate the longevity of the cellular immune response induced by vaccines.

To monitor the immune memory response, we developed a memory B cell FluoroSpot assay specific for pertussis antigens. This enabled us to detect antigen-specific memory B cells 6 months after the last vaccine dose, demonstrating that the minipig model can be used to monitor long-term immune memory responses. The number of IgG memory B cells specific for the pool of pertussis antigens was low, but the level is consistent with what has been reported in humans (46, 50). No difference was observed in circulating pertussis antigen specific memory B cells in DTaP and DTwP primed minipigs or human subjects several months post-Tdap boost. In baboons, DTwP priming resulted in significantly more longterm memory B cells than DTaP priming although these antigen specific memory B cells were detected at an order of 10^1^-10^2^/10^6^ PBMC (31, 46, 50).

Single-cell RNA-sequencing technology has been used to characterize immune cell responses by investigating the immune cell populations in various animal models, including pigs (51, 52), and in this study, to the best of our knowledge, for the first time in Göttingen minipigs PBMCs. Our objective was to assess the impact of the Tdap booster on PBMCs of minipigs primed with DTaP and to characterize the long-term immune memory response 6 months after the last vaccine dose, when T cell and memory B cell FluoroSpot assays were also performed. Major cell populations were characterized successfully based on their transcriptomic profile, and scRNAseq-based overall estimation of the major cell population frequencies was similar to what has been previously observed using cytometry-based methods in pigs (53–55). More importantly, low frequency populations (e.g., pDC) or those with a transitionary transcriptomic profile, such as the non-terminally differentiated γδ T cells were well-identified. Regarding the evaluation of long-term immune memory response, our scRNAseq analyses showed that the Tdap booster increased the numbers of T cells with an “activated” state after *in vitro* stimulation with a pool of pertussis antigens. It was accompanied by stimulation of the interferon pathway in the T cells and monocytes/macrophages, which is compatible with the IFN-γ production in PBMCs from DTaP-primed minipigs observed by FluoroSpot, even 6-months post-Tdap booster. Taken together, these results suggest that DTaP priming followed by Tdap boost induces a long-term memory T cell response against pertussis antigens. This memory response is probably reinforced by the activation of antigen presentation pathways in the monocyte/macrophage population, i.e., cells able to specifically stimulate T cells when exposed to pathogens. Altogether, T cell Fluorospot and scRNAseq provided complementary information in characterizing the cellular immune response. Indeed, our T cell FluoroSpot assay is sensitive in quantifying antigen-specific cytokine-secreting cells in PBMCs from pertussis vaccinated minipigs, therefore determining the Th response profile induced by vaccination. scRNAseq allowed a deep analysis of intracellular pathways involved in activation of the immune response at a single cell level. Such analysis could not otherwise be performed using a Fluorospot assay alone.

We also detected memory B cells using scRNAseq assays in our samples. The cell frequencies were similar irrespective of the vaccination received. However, these cells represented all memory B cells and not just *B. pertussis* specific memory B cells. A deeper analysis would be needed, either by increasing the total number of B cells, or by sorting B cells specific for pertussis antigens to characterize pertussis-specific memory B cells and by using new methods such as LIBRA-Seq (56).

Cell populations such as CD4^+^CD8^+^ T cells, known as activated and memory T cells, were not identified in the scRNAseq assay since the cells expressing these two markers were not in the same cluster (57). This may be due to differences observed between mRNA and protein expression, or to the fact that these cells have few differences in their transcriptomic profile, compared with CD4^+^ or CD8^+^ T cells. Currently, with the droplet-based scRNAseq assay, the genes studied represent the most expressed transcripts, i.e., only a partial view of the total transcriptome, and therefore may not always be sufficient to make a clear separation of cell types. Genes encoding cell surface markers were not always sufficiently detected to enable cell types sharing very close transcriptional patterns to be distinguished. The results that we presented could potentially be reinforced using a technology coupling detection of mRNA and protein expression such as CITE-seq or REAP-seq (58).

One of the limitations of minipig models is that although porcine IgG subclasses and their predicted functional properties have been previously described, based on their genomic sequences, their exact functionality in relation to human IgG subclasses is unknown (59). The route of maternal antibody transfer in pigs is different from that in humans, and therefore it is important to be cautious when interpreting results from the pig model for the influence of maternal antibodies (21, 22). Pigs are “high” γδT cell species and although porcine γδT cells share some similarities with human γδT cells, little is known about their biological function (8, 60). There are an increasing number of tools available to monitor porcine immune response. As shown in this study, commercial reagents are sensitive to assess cytokine-secreting Th-1 (IFN-γ), Th-2 (IL-4), Th-17 (IL-17) cells and antibody-secreting memory B cells by FluoroSpot. In our experience, antibodies for detecting antigen specific porcine total IgG, IgG1, IgG2, IgA in ELISA are also available. In addition, functional antibody assays such as pertussis toxin neutralization assay can be implemented. ELISA kits for cytokine quantification and fluorochrome-conjugated antibodies for identifying major immune cells such as CD4^+^ and CD8^+^ T cells, γδT cells, B cells, NK cells, monocytes and dendritic cells are also commercially available. Moreover, scRNAseq being a generic technology does not have species limitations. Regarding the scRNAseq data analysis, the pig genome is available and sufficiently annotated. However, reagents for the characterization of lymphocyte activation and differentiation are yet needed. It will also be important to have reliable multiplex cytokine detection methods for use in minipigs. Finally, the use of the minipig model for a combined evaluation of pertussis vaccine immunogenicity and protective efficacy, is limited by their resistance to *B. pertussis* infection from 4 to 5 weeks of age, and the fact that a *B. pertussis* challenge can only be done through an invasive intra-pulmonary *B. pertussis* inoculation (61, 62). Thus, vaccine efficacy could not be assessed in our study, particularly under an immunization schedule that mimicked the human pediatric priming, adolescent boosting pertussis vaccination scheme. Nevertheless, the protective immunity of other vaccines against their respective pathogens may be assessed in this preclinical model, as already described for example for Influenza (63, 64), *Chlamydia trachomatis* (65) or *B. parapertussis* (66). A combined immunogenicity and challenge model will be useful for identifying correlates and thresholds of immune protection.

In conclusion, our results demonstrated that this Göttingen minipig model can be used to characterize vaccine-induced immune responses, including IgG titers, functional antibodies, Th-1, Th-2, Th-17 profiles, memory B cells, and the longevity of the response. In addition, advanced analytical tools such as scRNAseq can be used with this model to investigate the characteristics of cell populations, study the interactions between immune components, and monitor the long-term memory response after vaccination. In addition to ethical and financial concerns when compared to NHPs, the minipig model displays particular advantages for vaccine evaluation. Notably, it is practical to evaluate a pediatric vaccine in neonatal minipigs due to their large litter size and ontogeny. Moreover, the magnitude and the kinetics of the response can be assessed in the same individuals, in a longitudinal study using immunization schedules similar to those used for humans, that are otherwise difficult to model in mice and NHPs. The onset, kinetics and longevity of the immune responses induced by DTwP and DTaP priming followed by Tdap boosting in the Göttingen minipig model were shown to be very similar to human immune responses. Therefore, our data, along with data from previous studies in pigs or minipigs support the use of pigs, particularly, Göttingen minipigs as a useful preclinical model for assessing the long-term immunogenicity of pertussis vaccines, and candidate human vaccines against other pathogens (45, 67, 68).

## Data Availability Statement

The datasets presented in this study can be found in online repositories. The names of the repository/repositories and accession number(s) can be found at: https://www.ncbi.nlm.nih.gov/geo/query/acc.cgi?acc=GSE164263.

## Ethics Statement

The animal study was reviewed and approved by Sanofi Pasteur's animal ethics committee.

## Author Contributions

CV and YL developed the study design. VC, EC, and CD developed the scRNAseq experimental study. CV, VG-B, and CD performed the experiments. CV, VG-B, VC, EC, CD, and YL participated in the data analysis and interpretation, drafted the manuscript and prepared the figures and revised the manuscript. All authors contributed to the article and approved the submitted version.

## Conflict of Interest

CV, VG-B, VC, EC, and YL are all employed by Sanofi Pasteur, Marcy l'Etoile, France. They hold shares/stock options as part of their remuneration package. CD was on a mission with Sanofi Pasteur from Kelly Services, Paris, France.

## Supplementary Material

Supplementary Figure 1 IFN-γ (Th-1), IL-17 (Th-17), and IL-4 (Th-2) secreting cells measured by FluoroSpot assay pre-boost (D77) on PBMCs stimulated with a pool of pertussis antigens (PTxd, PRN, FIM2/3) or heat-killed *B. pertussis* (hk*Bp*) for 24 h. Data for the DTwP (*n* = 7) and DTaP (*n* = 7) groups are shown as individual and geometric mean titers, after correction for background levels of cytokine secreting cells in the negative control wells (media alone). Data for the control group (TBS, *n* = 3) are shown as the geometric mean titer for all minipigs on D77, irrespective of the stimulation. ^*^*p*-value < 0.05 and ^***^*p*-value < 0.001 for statistical comparison of DTaP- and DTwP primed groups, NP = Not Performed because of too much non responders.

Supplementary Figure 2 Heatmap representing differentially expressed genes between **(A)** DTaP-primed and TBS groups both incubated with medium or **(B)** DTaP-primed group stimulated either with a pool of pertussis antigens or with medium. The differentially expressed genes (in rows) are represented for each major cell population (in columns). Down-regulated genes are indicated in blue (negative fold changes) whereas the upregulated genes are in red (positive fold changes).

## Abbreviations

DTwP: whole cell pertussis vaccine
DTaP: acellular pertussis vaccine
Tdap: booster acellular pertussis vaccine
SCs: secreting cells
MSD: MesoScaleDiscovery
HD: human dose
scRNAseq: single cell RNA sequencing
DT: diphteria toxoid
TT: tetanus toxoid
PT: pertussis toxin
PTxd: pertussis toxoid
PRN: pertactin
FIM: fimbria
FHA: filamentous hemagglutinin.
