# Supplementary figures and images for: Göttingen Minipigs as a Model to Evaluate Longevity, Functionality, and Memory of Immune Response Induced by Pertussis Vaccines
Source: Front Immunol. 2021 Mar 17;12:613810. doi: 10.3389/fimmu.2021.613810 (PMC8009978; doi:10.3389/fimmu.2021.613810)

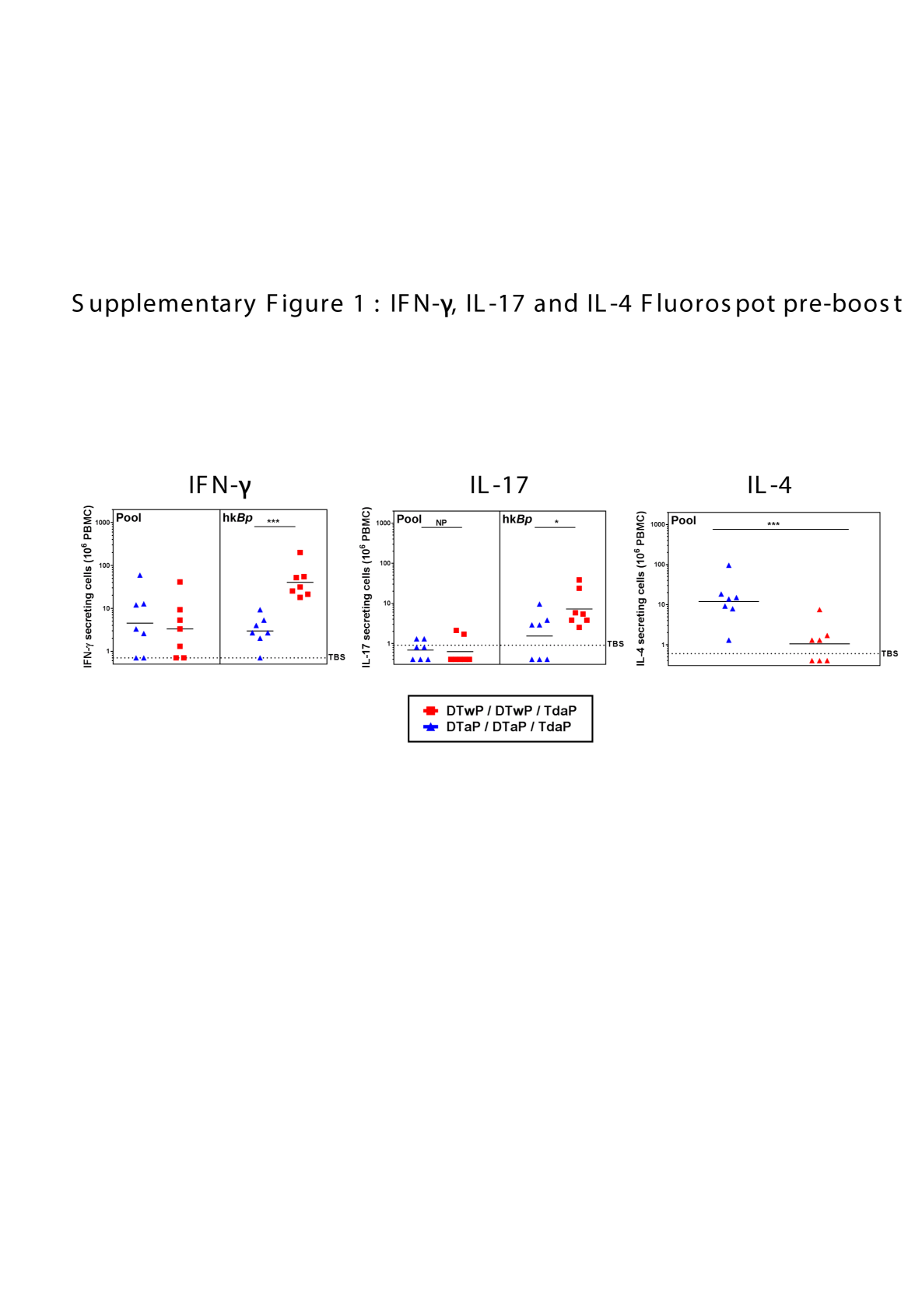

Supplement: Supplementary Figure 1 — IFN-γ (Th-1), IL-17 (Th-17), and IL-4 (Th-2) secreting cells measured by FluoroSpot assay pre-boost (D77) on PBMCs stimulated with a pool of pertussis antigens (PTxd, PRN, FIM2/3) or heat-killed B. pertussis (hkBp) for 24 h. Data for the DTwP (n = 7) and DTaP (n = 7) groups are shown as individual and geometric mean titers, after correction for background levels of cytokine secreting cells in the negative control wells (media alone). Data for the control group (TBS, n = 3) are shown as the geometric mean titer for all minipigs on D77, irrespective of the stimulation. *p-value < 0.05 and ***p-value < 0.001 for statistical comparison of DTaP- and DTwP primed groups, NP = Not Performed because of too much non responders. [file Presentation_1.zip › Supplementary Figure 1.tif]

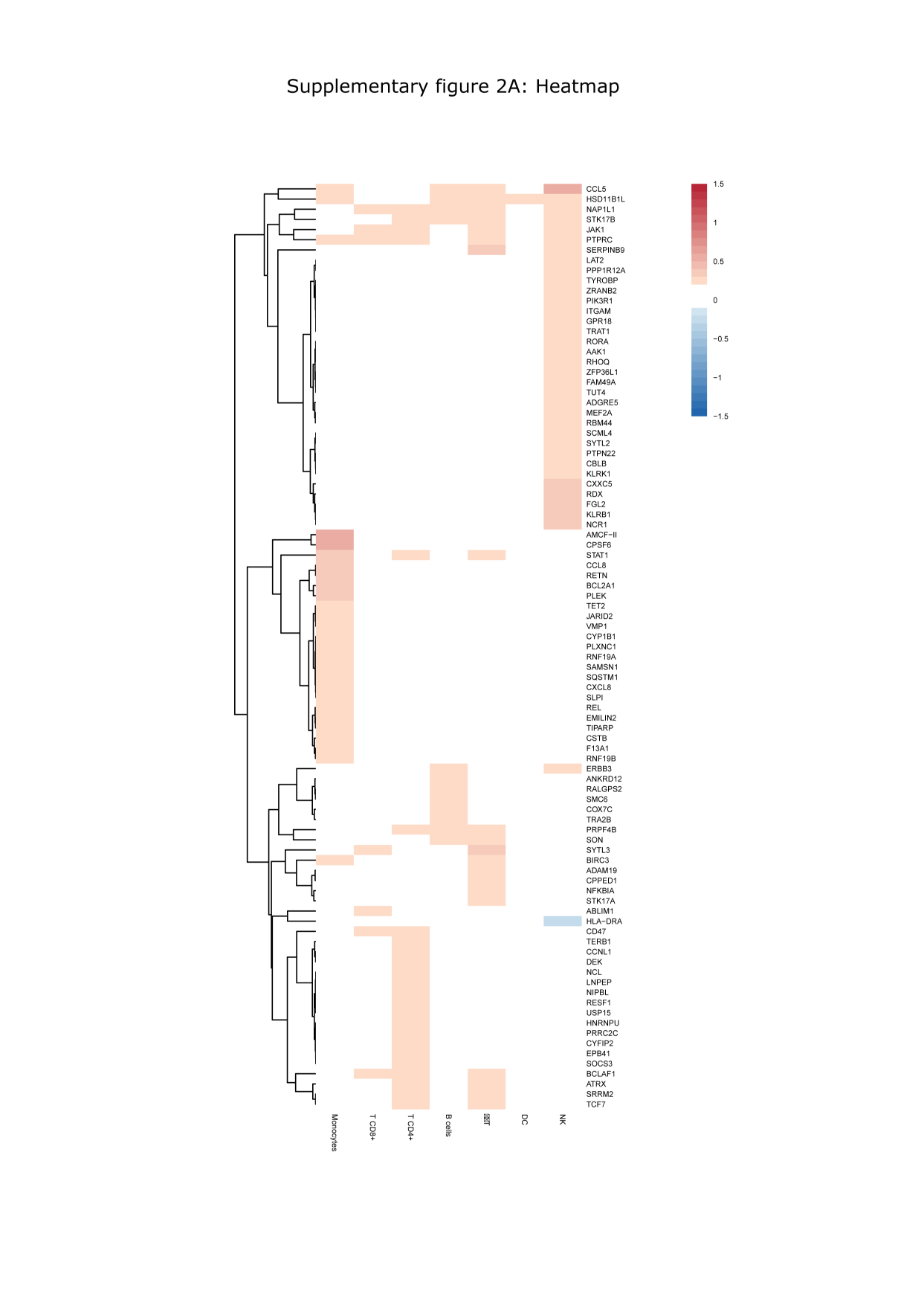

Supplement: Supplementary Figure 1 — IFN-γ (Th-1), IL-17 (Th-17), and IL-4 (Th-2) secreting cells measured by FluoroSpot assay pre-boost (D77) on PBMCs stimulated with a pool of pertussis antigens (PTxd, PRN, FIM2/3) or heat-killed B. pertussis (hkBp) for 24 h. Data for the DTwP (n = 7) and DTaP (n = 7) groups are shown as individual and geometric mean titers, after correction for background levels of cytokine secreting cells in the negative control wells (media alone). Data for the control group (TBS, n = 3) are shown as the geometric mean titer for all minipigs on D77, irrespective of the stimulation. *p-value < 0.05 and ***p-value < 0.001 for statistical comparison of DTaP- and DTwP primed groups, NP = Not Performed because of too much non responders. [file Presentation_1.zip › Supplementary Figure 2a.tif]

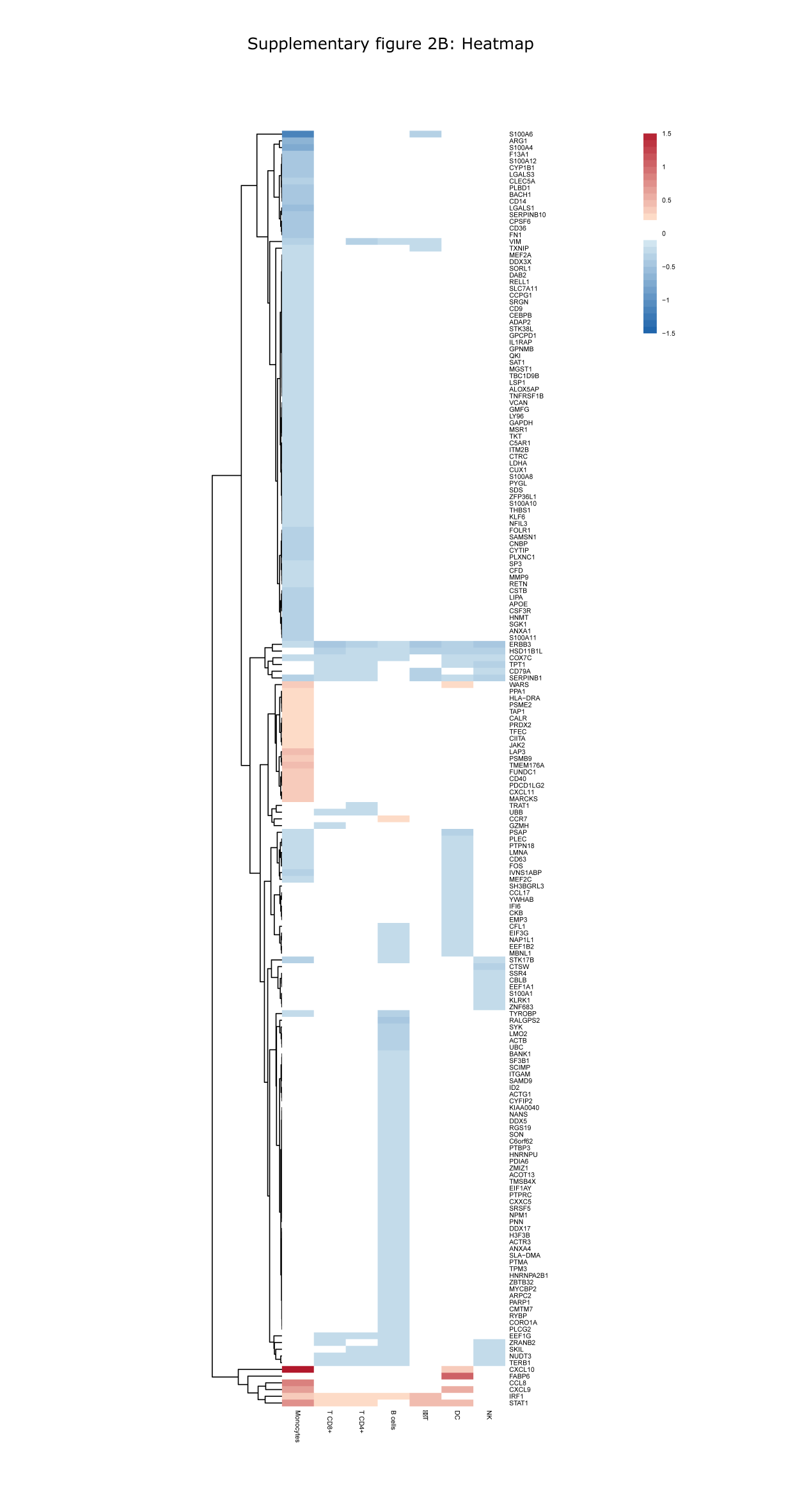

Supplement: Supplementary Figure 1 — IFN-γ (Th-1), IL-17 (Th-17), and IL-4 (Th-2) secreting cells measured by FluoroSpot assay pre-boost (D77) on PBMCs stimulated with a pool of pertussis antigens (PTxd, PRN, FIM2/3) or heat-killed B. pertussis (hkBp) for 24 h. Data for the DTwP (n = 7) and DTaP (n = 7) groups are shown as individual and geometric mean titers, after correction for background levels of cytokine secreting cells in the negative control wells (media alone). Data for the control group (TBS, n = 3) are shown as the geometric mean titer for all minipigs on D77, irrespective of the stimulation. *p-value < 0.05 and ***p-value < 0.001 for statistical comparison of DTaP- and DTwP primed groups, NP = Not Performed because of too much non responders. [file Presentation_1.zip › Supplementary Figure 2b.tif]
